# Describing the Development of a Health State Valuation Protocol to Obtain Community-Derived Disability Weights

**DOI:** 10.3389/fpubh.2019.00276

**Published:** 2019-09-27

**Authors:** Eunice Lobo, Lipika Nanda, Shuchi Sree Akhouri, Chandni Shrivastava, Roshan Ronghang, Geetha R. Menon, Ambarish Dutta

**Affiliations:** ^1^Indian Institute of Public Health- Bhubaneswar, Public Health Foundation of India, Bhubaneswar, India; ^2^National Institute of Medical Statistics, Indian Council of Medical Research, New Delhi, India

**Keywords:** EuroQoL, health state valuation, visual analog scale, disease burden, disability weights

## Abstract

For the prioritization of the allocation of national resources, estimating the burden of disease studies play a critical role. Hence the first Global Burden of Disease study conducted in the 1990s was done for this particular estimation. By the means of introducing disability-adjusted life year (DALY) metric, the burden of various diseases was calculated using disability weights (DWs)—a component of DALY. DWs are values that capture individuals' perception regarding the severity of diseases that involve valuation tools and health state descriptions. Various studies have been conducted over the past few decades to evaluate health states and derive disease-specific disability weights using Person-Trade off, Time-trade off, etc. However, use of these complex and cognitively demanding methods has been carried out in developed countries where the bulk of the populace is more educated. Few attempts have been made in low- and middle-income countries such as India, where not only the majority is less educated but also the social construction of diseases and health conditions are diverse. Therefore, due to the absence of methodological protocols of health state valuations for application at the community-level in the developing world, we attempted to systematically describe the procedure that can be used universally and cross-culturally for various health states. We began with the tentative selection of health states and health states valuation methods by conducting a meticulous literature review, followed by community exploration and medical consultations. This led to developing vignettes (clinical description) and 6D5L pictorial narrations (functional status description). Two field tests for checking the usability and refinement of the tools was done. Final consultation by an expert panel comprising of medical and non-medical professionals was held/conducted to finalize the health state labels and functional status profiles of each health state. The methodical approach provides a robust and thorough procedure for guiding researchers to implement health state valuation studies at community level.

## Introduction

For the prioritization of health research and public health initiatives, donors and countries need concrete and reliable data in terms of burden of diseases, especially for the allocative efficiency of resources for vulnerable groups and evaluating interventions (1). In the 1990s Murray et al. conducted the first Global Burden of Disease (GBD) study to estimate the burden of diseases—those that include the contribution of diseases to mortality and morbidity—for more than 100 health conditions along with disability-adjusted life year and disability weights. Disability weights (DWs) are assigned to diseases to compute their contribution to the burden, making disability weights an integral part of the burden estimation process (2, 3).

DWs are values obtained from individuals' perception on health states. The “health state valuation” underpins the disability weight estimation process, which includes a short, lay description of the health state with symptoms with or without description of functional status (EuroQoL 5D description system) (4, 5).

However, the GBD disability weights are not universal in nature as social and cultural contexts of health states were not accounted for in the GBD process (6). This is because the GBD valuers generally were educated professionals either from medical or health fields (7–9) and could easily participate in the cognitively demanding valuation methods (10–12). Although subsequent GBD and numerous other studies started including general population along with professionals as participants (9, 13–17), these studies were unable to capture the community-level perception of individual health states thus eliciting over- or under-estimation of health states.

Health behavior, environment, socio-economic status, education, and culture—factors broadly covered under the umbrella of social determinants of health—have started garnering a lot of importance lately. This has made the public views on health of paramount importance. People with different social status, education level and health states have different perceptions regarding health and thus it is imperative to develop interventions and policies with the understanding of their perspective on health (18). Therefore, the application of DWs from international studies, especially the developed countries for use in Low-middle income countries (LMICs) (4, 11, 13), are not suitable for use as a proxy for the local context especially in economically vulnerable communities' comprising of urban slums and rural populations. Strong evidence from empirical assessment done almost two decades ago in a village of South India (Andhra Pradesh) further emphasizes the need for community-derived DW suitable to India and its various regions (19). The Andhra Pradesh study highlighted that the community valuations of different health states do not all follow the same distribution as observed by the GBD study. However, owing to the small sample size and an exclusively rural context, the results were not generalizable to the national level and therefore required dire upgrading.

Furthermore, India has been experiencing rapid epidemiological and demographic shift owing to the increase in aging population and structural changes in disease patterns (20). These changes, alongside the changing social context for various health states within the community, that occurred over the past two decades needed to be explored. Hence, there arose a pressing need for a community-based health state valuation study (methodological pilot) to account for the changing societal perspectives across different Indian communities while estimating DWs. Therefore, a methodical and robust procedure was undertaken with an objective to estimate DWs at the community-level. Due to paucity of literature regarding health state valuation protocol, we attempted to develop a procedure that would be thorough to elicit DWs from community members of different locations, literacy levels, and socioeconomic strata. This paper describes the entire “Health State Valuation” process used for estimating the community-level DWs.

## Methods

### Scoping Review and Tentative Selection of Health States

#### Literature Search on Burden Diseases in India, and Various Health State Valuation Methods

A literature search for the burden of various health states in India was done using the data visualization tools and literature available from the Institute of Health Metrics and Evaluation (IHME) website. Additionally, research on the health state valuation methods used for deriving DWs from the year 1990 onwards was done using PubMed and Google Scholar.

The pioneering GBD study in 1996 valued health states using Person trade-off (PTO) (21) where medical experts were asked to make a choice between two groups of people—one with perfect health and the other with a specific health state of interest in order to allocate resources hypothetically (19). Furthermore, studies used alternative methods for health state valuation for instance Time trade-off (TTO), Standard gamble (SG), Paired comparison (PC), Visual analog scale (VAS), and Discrete choice experiment (DCE) (3, 11, 17). The TTO method involves trade-off between a short period lived in full health and fixed duration of time spent with disability to arrive at the disability weight for a given health state. Similarly SG requires the valuer to gamble between three choices—best (perfect health), worst (death), and an intermediate state of health with varying disabilities (19). The PC method provides various combinations of health states in pairs and the participants are required to select the healthier option based on their preference using multiple iterations (3). DCE requires participants to choose between two health states varying considerably, followed by drawing inferences based on random utility theory (22).

Since these methods are cognitively demanding, they may not be appropriate at population level especially in developing countries. Additionally, concerns about their time-consuming nature have been opined by Green et al. (10), Stouthard et al. (11), and Sung et al. (12). Moreover, literature hardly throws light on methods applicable for populations with lesser educational attainment, which further rationalizes efforts for refining methods to examine disability weights using a community lens.

#### Tentative Selection of Health States Based on Literature Search

Against the backdrop of the existing evidence above, a process to select the health states for our methodological pilot study was initiated. Data on DALYs of different health states was derived from the IHME database. A thorough literature search that was conducted suggested changes in the burden of various diseases in the country from the year 1990 to 2016, following the epidemiological and demographic shift. We found that although a substantial burden of communicable diseases has reduced, diarrheoa, lower respiratory infections, and tuberculosis continue to be ranked high among the leading causes of DALYs in India. The contribution of major non-communicable diseases (NCDs) that include cardiovascular diseases, chronic respiratory diseases, mental health, neurological disorders, neoplasms, and musculoskeletal disorders to the total disease burden has increased significantly over the last 20 years. The burden of diabetes is rising, and the DALY rate has increased four-fold, while stroke has moved seven positions upward to rank as the fifth leading cause of DALY in India. Additionally with the notable increase in road traffic accidents, road injuries features among the top 10 causes of DALYs (23). Hence, we shortlisted the above health states with the addition of locally relevant acute febrile illnesses (to ~50 health states).

### Finalization of Health States

#### Community Exploration (Urban Slums and Rural Villages)

Community exploration visits were conducted for understanding the community's knowledge, understanding, and social context regarding the shortlisted health states. Therefore, field visits were conducted in two villages of a nearby district—Dhenkanal—to capture the rural perspective, and in two urban slums of Bhubaneswar for understanding the views of the urban counterparts. We approached 15 community members with relatively low levels of education (no formal education to higher secondary level) from the villages and slums with a list of 10 health states picked from the list of 50 health states (malaria, dengue, tuberculosis, alcohol use disorder, low back pain, oral cancer, asthma, multiple depressive disorder, diabetes, and anemia), since discussing all health states would not be cognitively feasible for the participants.

Each interview session lasted for ~10 min and included probing questions on the awareness regarding diseases symptoms, care seeking behavior, and social stigma (if any) of the health states.

#### Consultation With Medical Professionals

Based on the learnings from the community exploration, we sought further guidance from medical professionals to finalize the health states and strengthen the justification of the included health states. Hence primary and secondary care government physicians from across the state were approached to understand the existing health scenario in different districts. Views of the specialists (orthopedician, psychiatrist, gastroenterologist, chest physician, and oncologist) from tertiary care hospitals were also taken into consideration to account for the same. The medical professionals were approached with a tentative list of 50 health states; the consultation included detailed account of the prevalent diseases and their signs and symptoms.

All participants' (community and medical professionals) discussions were audio recorded after obtaining consent. These discussions were further translated and transcribed by the study team and used for preparation of vignettes which will be described later.

After consultation with medical professionals and community exploration, the most relevant health states specific to the study locations were finalized for the community survey which amounted to 14 in number. Out of these, eight health states were to be valued by all participants irrespective of their gender and setting—tuberculosis, malaria, diarrhea, diabetes, osteoarthritis, asthma, quadriplegia due to stroke, and upper limb fracture due to road traffic accidents—while four health states were gender-specific—anemia and breast cancer (females) and alcohol use disorder and oral cancer (males). During the community exploration, we noted low levels of awareness of mental health in rural areas. Nonetheless, consultation with psychiatrists revealed the prevalence of depression and schizophrenia were notably high among rural and urban settings, respectively, hence the valuation of mental disorders in these two respective locations (Supplement 2).

### Tools for Describing Health States

#### Vignettes and 6D5L Description System

Using the information gathered from community visits as well as medical consultation, clinical description of health states called vignettes were prepared to describe the finalized 14 health states. Diagnostic and Statistical Manual of Mental Disorders−5 (DSM-5) criteria and International Classification of Diseases, Tenth Revision (ICD-10) classification were referred, where necessary, to enrich the vignettes. The vignette descriptions were similar to that of health state lay descriptions of GBD 2015 (clinical signs and symptoms characteristic of the given health states), with the addition of gender and age of the sufferer. Colloquial terms were incorporated wherever deemed necessary for better understanding of community members. After preparation, the health state vignettes were translated from English to Odia language by a native Odia speaker.

Health states with description of symptoms along with functional status are highly effective in health state valuation studies (24–26). Thus, after the preparation of health state vignettes, a modified EQ-5D+ (EuroQol) (27) instrument was used alongside the vignettes to further describe the health state's functional status. EQ-5D+ includes a structured approach where each health state is described in terms of dimensions and severity levels within each dimension. An adaptation of EQ-5D+ was used by the Andhra study (28), where six dimensions with five levels each were considered and referred to as 6D5L description system. The pictorial description system including six dimensions—mobility, self-care, usual activity, pain/discomfort, anxiety/depression—and cognition with five varying levels of severity of the Andhra study was used initially.

### Tools for Valuation of Health States

#### Card Sort and Visual Analog Scale

Cognitively demanding techniques such as the SG, TTO, and PTO which have been used extensively by previous disability weights studies require a certain level of education to comprehend and use (29). Therefore, these methods are more complicated than the visual analog scale (VAS) and the modified card sort (CS) methods that were used in our study. Moreover, any valuation process irrespective of the technique used requires participants (hereafter referred to as valuers) to visualize the entire description of health states. This may require multiple deliberations since these tasks require the valuer to understand the tools used and spend enough time to carry out the complex tasks of valuing each health state. Additionally, the tools need to be universal in application such that cross-cultural differences can be captured and evaluated (30).

The VAS method uses a continuous graduated line segment, one end labeled as “death” and the other labeled as “perfect health” ranging from 0 to 100. It allows the user to rate a particular health state on between the mentioned anchor points. The method is cognitively simple to use (31) and can be conducted at the community level for 10–12 health states at one occasion and as a tool to quantify subjective phenomena is sufficiently valid and reliable (32). The process requires the valuer to assign a score to each health state based on their understanding. The score obtained is then used to calculate disability weights as per the formula below,

(1) DW=1−(VAS/100)

However, in our study the VAS procedure was preceded by the modified CS process which is not only a validation tool but also a “warm up” for the entire valuation process. It further helped to strengthen the process of arriving at the final VAS scores through various iterative rounds.

Further prior to card sorting, in order to orient the valuer to the entire process of ranking various health states, each valuer was asked to score their own health state initially. Then each health state description was read aloud and the valuer was asked to order the health states from 1 to 11 referred to as “least severe” health state to “most severe” health state, respectively. Rank order of each health state was recorded. The valuer then moved on to valuation by visual analog scale method. Here the valuers were instructed to assign values or scores according to their understanding of the magnitude of severity of the health state. A picture of a happy face near “100” on one side and a picture of a sad face near “0” further helped focus the valuer to the direction of severity (Supplement 1). At the end of both exercises, the card sort rank and VAS scores were checked for concordance, by the investigator. In instances where according to the investigator the values and ranks did not correspond, the valuer was requested to review his/her responses through iterations.

The interview schedule used for valuation had three sections: (1) socio-demographic profile of valuer; (2) “own health state” valuation using VAS; (3) valuation of individual health states selected for the study using CS and VAS, along with own or contact history.

### Reduction of 6D5L to 6D3L, Preparation of Labels, and 6D3L Description System

After the tools were developed, in order to check the usability two field tests were conducted using contrasting populations—urban educated elite and rural population with relatively less education. The five urban participants were graduate level and above, including academicians, IT professionals, and research associates, while the five rural participants were homemakers, casual laborers, and unemployed with no formal to primary level education. The purposive nature of sample was done to learn the differences between the two distinctly different groups and accordingly modify the tools to be as universal in application as could possibly be.

The urban field tests were conducted in Bhubaneswar, while the rural interviews were conducted in a tribal pocket of a nearby district, Angul. After explaining the study details, written consent was obtained from willing participants. The consent included participation in the interview and permission to publish anonymized information in reports/journals, etc. Each interview was done in the language (Odia, Hindi, and English) and location of preference (house and workplace) of the valuer. The interviews' duration were ~60–90 min.

As anticipated, a striking difference between both groups was observed. This included understanding long, complicated sentences (vignettes) among rural valuers, and the suggestion of the inclusion of duration and treatment status of health state for better valuation by urban valuers. However, one of the most prominent findings that emerged from this exercise was that both groups struggled to distinguish between the five severity levels. Additionally, for CS, difficulty in ranking the health states between the two end points was observed. Similarly, assigning a single score for the VAS exercise on a continuous scale was also challenging due to the inability to understand the difference between least, intermediate, and most severe health states. Moreover, problems in comprehending and relating to the drawings of each dimension especially anxiety/depression and cognition was noted.

Thus, two psychiatrists were sought separately to modify the appropriate severity levels and the pictorial narrations for anxiety/depression and cognition dimensions, while severity levels of the remaining four dimensions were reduced based on the study team consensus. Therefore, the levels of all the six dimensions were reduced from five to three as seen in Table 1.

**Table 1:** The 6D3L description system developed after field tests and medical consultations.

**Dimension**	**Dimension description**	**Severity level**
Mobility	Getting around in the community, Walking, climbing stairs, etc.	1 -No problems walking about 2 -Some problems walking about 3 -Confined to bed
Self-care	Bathing, cleaning, washing, toileting, etc.	1 -No problems with self-care 2 -Some problems washing or dressing self 3 -Unable to wash or dress self
Usual activity	Performance of usual role activities such as working at a job, housework, child care, volunteer work, etc.	1 -No problems with performing usual activities 2 -Some problems with performing usual activities 3 -Unable to perform usual activities
Pain/Discomfort	Subjective feeling of bodily distress of discomfort	1 -No pain or discomfort 2 -Moderate pain or discomfort 3 -Extreme pain or discomfort
Anxiety/Depression	Negative psychological states including anxiety, depression, behavioral emotional control, loneliness, etc.	1 -Not anxious or depressed 2 -Moderately anxious or depressed (social isolation and loss of appetite) 3 -Extremely anxious or depressed (suicidal ideation)
Cognition	Cognitive problems, such as forgetfulness, difficulty in concentrating, loss of tempero-spatial orientation, etc.	1 -No problems in cognition 2 -Some problem with memory and concentration 3 -Severe problem in cognition (loss of tempero-spatial orientation)

Additionally, since vignettes were unable to capture the valuer's attention for long, the use of labels was finalized. Labels were short descriptions of health states that included clinical symptoms relating to intensity of the health states, treatment status, and duration (where necessary) in simple vocabulary. The psychiatrists and relevant medical experts were consulted at length to finalize the same, especially for use of easy-to-understand jargons.

**Figure 1:**
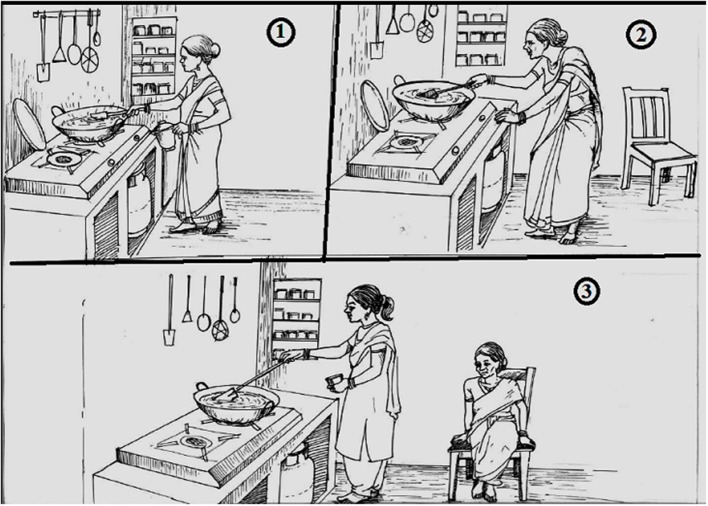
Usual activity (third dimension) with the example of a homemaker, respectively with the three levels of severity: i.e., (1) no problem, (2) mild/moderate problem, and (3) severe problem. The figure depicts the third dimension (usual activity) and third severity level (severe problem) of a homemaker, who is unable to perform her usual activity of cooking and requires help.

Therefore, the study team decided to further refine the tools by developing a new set of pictorial narrations that incorporated the changes based on the learnings from the field and medical experts for the reduced 6D3L description system. Hence, an artist was commissioned from a renowned fine arts university to develop a new set of pictures describing the three severity levels under each of the six dimensions, hence 18 pictures to start with. Then these 18 pictures were further developed for a male farmer (rural), a male shopkeeper (urban), and a female homemaker as seen in Figure 1, for easier comprehension by the most commonly occurring valuers as anticipated. The local social context relevant to respective settings were depicted through the pictures. Several drafts of pictures were drawn by the artist and shared with the study team until 54 pictorial narrations were finalized (three sets of 18 pictures each).

To overcome the issue of ranking and assigning scores, the study team decided to divide the rank order into two parts for ease of understanding. Thus, during CS, the description of each health state was read out to the valuers in random order by the interviewer, who would then be instructed to rank their preference between 1 and 5 for less severe health states (according to their choice) and 6–11 for more severe. Similarly, for assigning scores on a scale from 0 to 100, valuers would be explained the end points—“100” signified “perfect health” while “0” was “near death.” The VAS scale thus had the addition of pictures of faces with varied expression for differentiation of severity of the scale. Multiple iterations would be conducted until the valuer's response for each health state was final.

### Connecting the Labels and 6D3L Pictorial Narrations—“Adding Pictures to Words” for Each Health State

Prior to the community survey, health state-specific functional status profiles were finalized that would be used along with the labels to describe individual health states at the community level.

**Figure 2:**
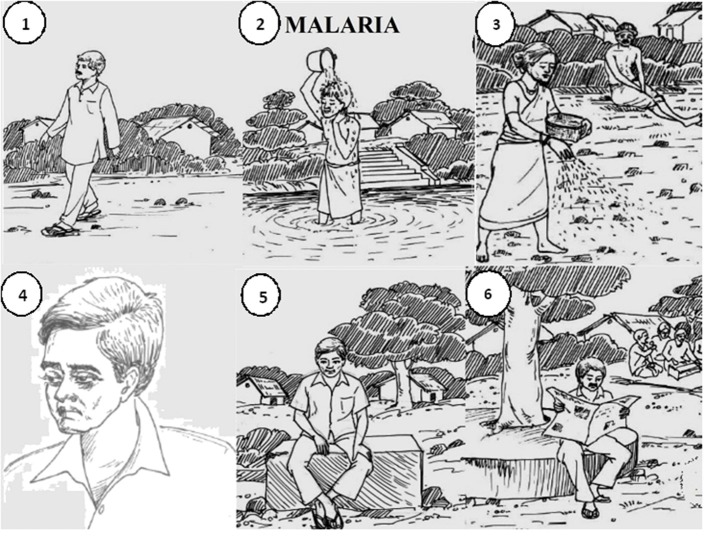
6D3L pictorial narration of Malaria with: (1) no problem in mobility, (2) no problem in self-care, (3) severe problem in usual activity, (4) mild/moderate pain/discomfort, (5) no anxiety/depression, and (6) no problem in cognition. The figure depicts the pictorial narration of Malaria as developed by expert panel and study team members, where the severity level of each dimension has been appropriately defined.

In this step, each health state-specific functional status profile was culminated through the inputs of panel experts using the 6D3L pictorial narration. After understanding the description of each health state label, the panel experts selected one appropriate level from each dimension so that each health state would be pictorially represented by six dimensions and one level of each dimension. Figure 2 shows the pictorial narration of a male Malaria patient describing his functional status profile while suffering from the disease.

The expert panel composition was as follows:

- Medical—Physicians from primary and secondary care public sector across Odisha, and varied specializations (Orthopedics, Psychiatry, Community Medicine, Pulmonary Medicine, Neurology) and staff nurses from tertiary care private sector hospitals in Bhubaneswar.
- Non-medical or lay—Academicians from a public university and private college and IT professionals, administrative officers from the private sector in Bhubaneswar.

Any suggestions by experts regarding change in any descriptions (labels) were taken into account and then modified. A summary of the expert panel recommendations was compiled to construct health state-specific functional status profiles (expert panel profile). Further through our knowledge and understanding of the health states, we selected an appropriate level from each dimension to develop the health state-specific functional status profile (provisional profile). These profiles were then matched with the expert panel profiles.

Thus, through harmonization of recommendations, six dimensions with health state-specific severity level of the 14 individual health states' functional status were represented pictorially.

Description of the profiles and labels of each health state was prepared in English and translated into Odia and Telugu languages by native speakers of respective languages.

## Anticipated Results

For the community survey a researcher booklet was prepared that contained the health state labels in Odia and Telugu languages, 6D3L pictorial narrations of each dimension and severity level, and 6D3L pictorial narration profile of individual health state for a farmer, shopkeeper, and homemaker each.

In the study we used VAS or obtaining values used for computing community derived disability weights, which is a tested and validated methods used since the early 1990s. We made use of the visual analog scale along with card sort as the valuation tool instead of the alternative methods as person trade off, time trade off as well as the recent paired comparison or discreet choice experiments due to the easy comprehension by the different community members. We strongly believe that the simple and ease-of-use features that the used tools offer make the task of valuation cognitively less demanding on the valuer and hence selected the appropriate choice not only for vulnerable but also rural populations to understand their health preferences. The added combination of VAS scale coupled with card sort exercise with multiple iterations further improved the sensitivity of the tool in our study.

Our protocol has been used at the intended community-level thus enabling us to successfully record the health state valuations of the urban and rural communities across two diverse states in India—Odisha and Telangana. The results have not been incorporated in the present piece of work as it is beyond the scope of the paper.

## Discussion

Our study used Visual Analog Scale as the tool for obtaining values required for computing community-derived disability weights—a tested and validated method since the early 1990s. The deliberate combination of VAS with card sort method, rather than the alternate person trade-off, time trade-off or the recent paired comparison or discreet choice experiments, ensured easier comprehension by community members across different sections of society and location. We strongly believe through our pilot initiative that the simple and ease-of-use features the tools offer make the task of valuation cognitively less demanding on the valuer and thus an appropriate choice not only for the less educated but also rural populations for understanding their health preferences. The additional multiple iterations further improved the sensitivity of the tool in our study.

Our protocol has been successfully used at the community-level to record the health state valuations of the urban and rural communities across two diverse states in India—Odisha and Telangana. The process that also involves multiple iterative regarding the responses of the participants enabled us to obtain precise values of the individual health preferences. The results of the same are beyond the scope of this paper, as they will be independently presented.

Furthermore, as per our knowledge this is the first methodological protocol developed that can be used for deriving disability weights at the community-level in low-middle-income countries. The approach employed by us for the preparation and implementation of health state description and valuation tools has been done in meticulous detail. Moreover, this well-designed, pre-tested, systematic protocol has been prepared for use across various locations, cultures, and even countries to explore differences in health state values. The robust nature of the protocol can further be applied to update the existing methods of obtaining disability weights, where the inclusion criteria need not be restricted to the educated elite. The principle of our methodological approach along with steps conducted to arrive at rigorous and thorough tools will further guide researchers that can implement studies at community-level. The relative dearth of population-level studies, especially in India with varying levels of education, will facilitate capturing perception-related disability weights since these in turn will play a significant role in policy decisions for resource allocation in health care and prevention.

## Data Availability Statement

The datasets generated for this study are available on request to the corresponding author.

## Ethics Statement

The studies involving human participants were reviewed and approved by ethical approval was obtained from the Indian Institute of Public Health, Bhubaneswar vide IEC no. IIPH/IEC/2017/20. The patients/participants provided their written informed consent to participate in this study.

## Author Contributions

EL, SA, CS, and RR were involved in the conception and design. EL and SA were involved in drafting of paper and revising the draft. AD, LN, and GM were involved in critical analysis of the paper. All the authors have approved the final version of the article submitted.

### Conflict of Interest

The authors declare that the research was conducted in the absence of any commercial or financial relationships that could be construed as a potential conflict of interest.
