# Supplementary material for: Describing the Development of a Health State Valuation Protocol to Obtain Community-Derived Disability Weights
Source: Front Public Health. 2019 Sep 27;7:276. doi: 10.3389/fpubh.2019.00276 (PMC6798035; doi:10.3389/fpubh.2019.00276)

Card Sort and VAS Scoring

**Own health state**	Mobility	Self care	Usual activity	Pain/ discomfort	Anxiety/ depression	Cognition	VAS Score

CS RANK	Health State	History	Code	VAS Score
1		a		
		b		
2		a		
		b		
3		a		
		b		
4		a		
		b		
5		a		
		b		
6		a		
		b		
7		a		
		b		
8		a		
		b		
9		a		
		b		
10		a		
		b		
11		a		
		b		

Visual Analogue Scale

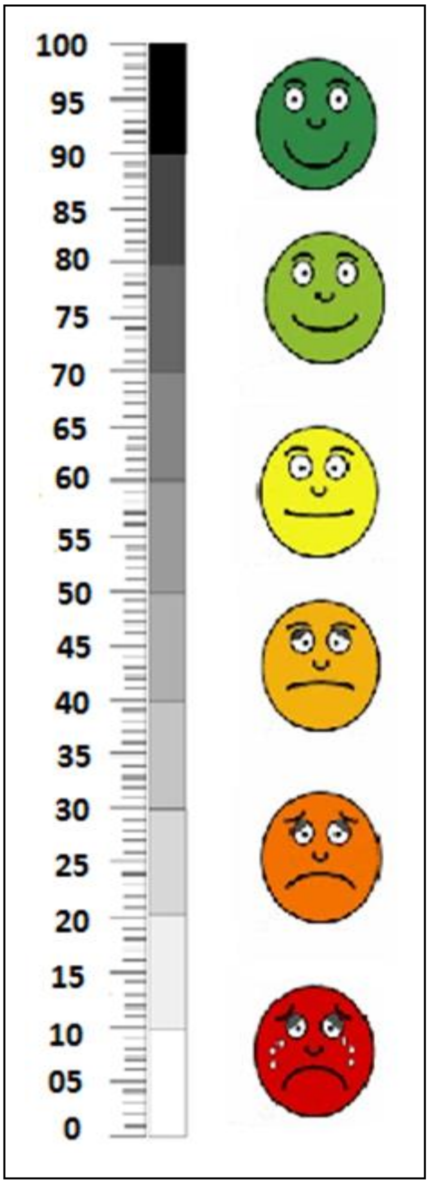

Supplement: Supplementary file 1 [file Data_Sheet_1.PDF]
