# Supplementary material for: Describing the Development of a Health State Valuation Protocol to Obtain Community-Derived Disability Weights
Source: Front Public Health. 2019 Sep 27;7:276. doi: 10.3389/fpubh.2019.00276 (PMC6798035; doi:10.3389/fpubh.2019.00276)
Supplement: Supplementary file 2 [file Data_Sheet_2.PDF]

Supplement 2: List of Health states and the type of valuer based on gender and location

S.no	Health state	Valuer			
		Male Urban	Male Rural	Urban Female	Female Rural
1	Tuberculosis	✓	✓	✓	✓
2	Diabetes	✓	✓	✓	✓
3	Malaria	✓	✓	✓	✓
4	Diarrhoea	✓	✓	✓	✓
5	Upper limb fracture due to RTA	✓	✓	✓	✓
6	Asthma	✓	✓	✓	✓
7	Osteoarthritis	✓	✓	✓	✓
8	Quadriplegia due to stroke	✓	✓	✓	✓
9	Anaemia			✓	✓
10	Breast cancer			✓	✓
11	Alcohol use disorder	✓	✓		
12	Oral cancer	✓	✓		
13	Depression		✓		✓
14	Schizophrenia	✓		✓	
**TOTAL**		**11**	**11**	**11**	**11**
